# A multidimensional toolkit for elucidating temporal trajectories in cell development *in vivo*

**DOI:** 10.1242/dev.204255

**Published:** 2024-12-18

**Authors:** Masahiro Ono, Tessa Crompton

**Affiliations:** ^1^ Department of Life Sciences, Imperial College London, London SW7 2AZ, UK; ^2^ Great Ormond Street Institute of Child Health, University College London, London WC1N 1EH, UK

**Keywords:** Multidimensional analysis, Canonical correspondence analysis, Fluorescent Timer, Developmental trajectory analysis, T-cells, Thymus, Mouse

## Abstract

Progenitor cells initiate development upon receiving key signals, dynamically altering gene and protein expression to diverge into various lineages and fates. Despite the use of several experimental approaches, including the Fluorescent Timer-based method Timer-of-cell-kinetics-and-activity (Tocky), analysing time-dependent processes at the single-cell level *in vivo* remains challenging. This study introduces a novel integrated experimental and computational approach, using an advanced multidimensional toolkit. This toolkit facilitates the simultaneous examination of temporal progression and T-cell profiles using high-dimensional flow cytometric data. Employing novel algorithms based on canonical correspondence analysis and network analysis, our toolkit identifies developmental trajectories and analyses dynamic changes in developing cells. The efficacy of this approach is demonstrated by analysing thymic T cells from Nr4a3-Tocky mice, which monitor activities downstream of the T-cell receptor (TCR) signal. Further validation was achieved by deleting the proapoptotic gene *Bcl2l11* in Nr4a3-Tocky mice. This revealed dynamic changes in thymic T cells during cellular development and negative selection following TCR signalling. Overall, this study establishes a new method for analysing the temporal dynamics of individual developing cells in response to *in vivo* signalling cues.

## Teaser

**Summary:** A novel toolkit, utilising Tocky-canonical correspondence analysis, enables the identification of developmental trajectories *in vivo* through flow cytometric analysis using Fluorescent Timer proteins, elucidating temporal trajectories of thymic T-cell development.

## INTRODUCTION

In multicellular organisms, immature cells initiate developmental processes upon receiving key signals, changing their gene expression and cellular phenotype, and thereby producing a variety of cell lineages (Holguera and Desplan, 2018). A longstanding challenge in developmental biology has been understanding the temporal control of transcription of genes that play central roles in regulating developmental processes (Raimundo and Levine, 2023). The temporally dynamic control of gene activity is crucial for regulating fates in developing cells (Voortman et al., 2022). The timing of gene expression is tightly regulated by the coordinated activities of gene regulatory regions, which in turn control cellular development and differentiation (Raimundo and Levine, 2023). Therefore, experimental measurement of the passage of time in individual cells since the transduction of a key developmental signal should help to elucidate dynamic cellular and molecular mechanisms during cell development. However, a major difficulty in analysing the temporal dynamics of cell development is that a population of progenitor cells does not receive a developmental cue in a synchronised manner.

Time course analysis can allow for the examination of the timing of gene expression following key signalling for development in cell cultures (Solanki et al., 2020) and in small animals such as *Caenorhabditis elegans* using microscopy (Aymoz et al., 2018; Perales et al., 2014). However, this approach cannot be directly applied to the analysis of developing cells *in vivo* in larger animals, such as mice and humans. First, the time and temporal resolution of *in vivo* microscopy are insufficient for investigating cellular development of tissues in mammals, which are larger and include a wide range of cell types. Although single-cell analyses, such as flow cytometry and RNA sequencing (RNA-seq), can provide insights into the regulation of developmental processes, signal transduction inherently has a stochastic component *in vivo* (Lancaster et al., 2019), which hampers accurate measurements of the timing following key developmental cues. Therefore, experimental measurement of the passage of time in individual cells since receiving a developmental signal should advance understanding of the temporally dynamic nature of the mechanisms that underlie cell development *in vivo*.

Recent advancements in single-cell technologies have revolutionised the analysis of time-dependent changes in cells *in vivo*. Pseudotime analysis, a popular method for trajectory analysis, can help to elucidate gradual cellular changes during development or following activation. This analysis is performed within a reduced-dimensional space, typically using principal component analysis (PCA) or uniform manifold approximation and projection (UMAP), and is applied to single-cell RNA-seq data (Bradley et al., 2018; Kalfaoglu et al., 2020; Tan et al., 2021). Pseudotime analysis has also been applied to high-dimensional flow cytometric data (Guilliams et al., 2016), although the application is currently limited due to uncertainties about its interpretability. Crucially, pseudotime analysis does not depend on direct experimental measurements of time. Instead, it analyses the similarity of gene expression profiles among single cells within these reduced dimensions. By doing so, pseudotime constructs a trajectory of developmental stages, ordering cells based on their gene expression similarity, which does not necessarily reflect actual temporal data or indeed provide a measure of the duration of the developmental process in real time (Becht et al., 2019). Consequently, it is essential to develop new experimental methods that can directly measure time to understand the developmental trajectories of cells *in vivo* more accurately.

The ability to measure the passage of time after signal transduction as a developmental cue was our motivation to develop the new analysis method ‘Timer-of-cell-kinetics-and-activity’ (Tocky), which uses Fluorescent Timer as a reporter gene to analyse temporal changes in transcriptional activities (Bending et al., 2018a). However, the adaption of Tocky to investigation of temporal dynamics of *in vivo* cell development at the single-cell level requires an established method for analysing high-dimensional, flow cytometric Tocky data. The primary issue here is the significant imbalance in data: the multidimensional marker profile data typically exhibit large variances, whereas the dimension of Timer fluorescence is limited to only two. This disparity makes it difficult to integrate the time-dependent changes indicated by Timer fluorescence progression with the developmental steps defined by marker profiles, complicating the understanding of cellular development over time.

To cross-analyse Timer fluorescence data with multidimensional marker profile data, canonical correspondence analysis (CCA) is a promising approach. CCA was originally developed in ecology for understanding the influence of environmental gradients (e.g. pollution) on species distribution and abundance in an environment (ter Braak, 1986). We previously adapted CCA as a unique dimensional reduction method with a focused analysis on defined biological processes in a data-oriented manner using transcriptome data from microarray (Ono et al., 2013), bulk RNA-seq (Ono et al., 2014), and single-cell RNA-seq (Bradley et al., 2018). CCA analysis of transcriptomes has successfully revealed dynamic processes in thymic T-cell development (Furmanski et al., 2013; Saldaña et al., 2016; Sahni et al., 2015; Sinclair et al., 2015; Solanki et al., 2018, 2020; Mengrelis et al., 2019; Lau et al., 2021), differentiating helper and regulatory T cells in the periphery (Ono et al., 2014), T-follicular helper cells in diabetic animals (Kenefeck et al., 2015), tumour-infiltrating T cells from malignant melanoma patients (Bradley et al., 2018), and the leukaemic transformation of bone marrow cells (Ono et al., 2013) and of T cells by retroviral infection (Tan et al., 2021). However, to date, no attempt has been made to apply CCA for flow cytometric data, including Timer fluorescence data.

In this study, we have adapted CCA to high-dimensional flow cytometric data from Tocky mice to reveal temporally dynamic changes in developing cells, using T-cell development following T-cell receptor (TCR) signal transduction as a model system to show proof of principle of our approach. The new toolkit allows experimental measurement of time information in individual T cells during their development. To demonstrate the ability of this new computational tool, we used Nr4a3-Tocky to analyse the temporal dynamics of TCR-dependent processes following TCR signalling (Bending et al., 2018a). During T-cell development, TCR signalling acts as a developmental cue as well as instruction for lineage diversification in developing thymic T cells (Irla, 2022). Importantly, developing thymic T cells that receive strong TCR signals are deleted by negative selection via the proapoptotic gene *Bcl2l11* (also known as *Bim*) (Hojo et al., 2019). Thus, the deletion of the *Bcl2l11* gene in the Nr4a3-Tocky background delays apoptosis by negative selection and thereby permits analysis of thymic T cells undergoing negative selection.

## RESULTS

### Rationale and strategies for revealing temporal trajectories in developing cells

**Fig. 1.**
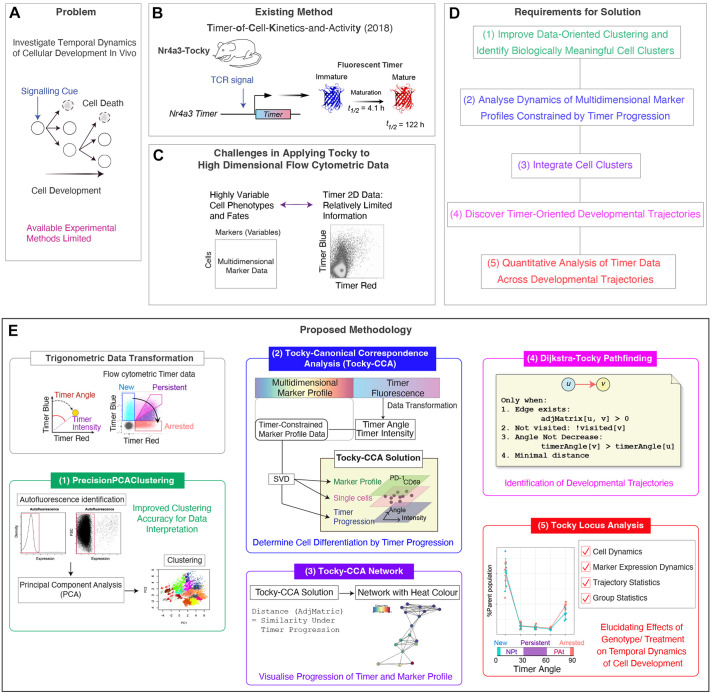
**Rationale and strategies for investigation of temporal trajectories in developing cells.** (A) The study aims to address to analyse temporal dynamics of cellular development *in vivo* and the limitation of available methods. (B) The existing Tocky method. Developing cells in Tocky mice will activate Timer transcription upon receiving TCR signalling as a developmental cue (transcription dynamics), which leads to the expression of Fluorescent Timer protein. The maturation of the Fluorescent Timer chromophore occurs with a maturation half-life 4.1 h. The mature Red protein decays slowly, with a decay half-life 122 h (protein dynamics). (C) Marker expression data are multidimensional and cell phenotypes and fates are highly variable in developing cell samples. In contrast, Timer fluorescence data are two-dimensional only, and the imbalance of dimensions poses a significant challenge. (D) Five requirements for solving the current problem are shown. (E) A flowchart of the new package ‘TockyDevelopment’. Data preprocessing consists of Timer fluorescence in Timer-expressing cells measured by flow cytometry to analyse fluorescence dynamics. Here, the existing Tocky method can perform trigonometric transformation of Timer Blue and Red fluorescence data into Timer Angle and Intensity. Timer Intensity is a distance (norm) between the origin and each cell, while Timer Angle is the angle from the Blue axis. Timer Angle is defined as Tocky Time in the study of developmental cell biology and is categorised into the five Tocky loci as defined by the degrees of angle (in parentheses): New (0°), Persistent (30-60°), Arrested (90°), and the regions in between [New-to-Persistent (NPt) 0-30°, and Persistent-to-Arrested (PAt) 60-90°)] (Bending et al., 2018a). PrecisionPCAClustering (1) combines autofluorescence identification, PCA, and k-means clustering. Tocky-Canonical Correspondence Analysis (Tocky-CCA) (2) is a newly developed algorithm that enables a balanced cross-analysis of multidimensional marker profile and Timer fluorescence data, using the projection of the marker profile onto Timer Angle and Intensity data and subsequent singular value decomposition (SVD). The solution includes three layers: marker profile, single-cell, and Timer progression layers, which are cross comparable to each other. The Tocky-CCA Network (3) consists of processing of the Tocky-CCA output for adjacent matrix (AdjMatrix), which represents similarities such as distance between cells in the CCA space. Dijkstra-Tocky pathfinding (4) is the newly implemented algorithm, which enables identification of a unique path across the Tocky-Network between the source and destination nodes using Timer Angle. Tocky locus analysis (5) is a quantitative analysis method that categorises Timer Angle into the five loci, enabling statistical analysis. High-dimensional flow cytometric Tocky data from developing Nr4a3-Tocky thymocytes are analysed through a series of canonical analyses. Timer fluorescence data are processed by trigonometric transformation, while non-Timer fluorescence data are analysed by a novel multidimensional clustering method involving definition and exclusion of autofluorescence, PCA, and k-means clustering. Processed data from the two branches of analyses are analysed by CCA to understand the dynamic changes of multidimensional cellular profiles as Timer progresses. The CCA output is used for Tocky Temporal Network Analysis, which allows topological analysis and lineage discovery of dynamically changing cells. Meanwhile, Tocky time progression analysis by Timer Angle density plot reveals the average temporal sequence of thymocyte development. Finally, Tocky-time analysis reconstructs the *in vivo* temporal dynamics of marker expression and reveals features of each cell lineage, which is facilitated by quantitative analysis of cluster dynamics.

Our study aims to address a long-standing problem in developmental cell biology and molecular biology: how to investigate the temporal dynamics of cellular development *in vivo* (Fig. 1A). In developing tissues, immature cells initiate their development in response to key signalling, leading to cell proliferation and diversification into various developmental trajectories. Despite the clear conceptual understanding of these processes, the *in vivo* experimental quantification and analysis of these cellular events remains significantly constrained by the limitations of existing experimental methodologies.

The Nr4a3-Tocky transgenic model incorporates the Nr4a3-Timer reporter, which initiates transcription of the Timer gene (*Fast-FT*) in response to TCR signalling (Fig. 1B). Upon translation, the Timer protein possesses an immature chromophore that emits blue fluorescence. This chromophore spontaneously and irreversibly matures into a chromophore with mCherry-type red fluorescence (Subach et al., 2009). We previously determined the kinetic parameters of this system: the Timer Blue protein exhibits a maturation half-life of 4.1 h, and the mature Timer Red protein has a decay half-life of 122 h (Bending et al., 2018a). Consequently, Timer fluorescence in Nr4a3-Tocky T cells captures temporal information from the onset of *Nr4a3* gene transcription, triggered by TCR signalling. Given that TCR signalling is crucial in determining the fate of developing T cells, the Nr4a3-Tocky model provides a valuable tool for elucidating the temporal sequence of T-cell development in response to this key signalling pathway.

However, analysing data from Tocky systems using high-dimensional flow cytometry presents substantial challenges (Fig. 1C). During development, cell phenotypes and fates exhibit high variability, resulting in marker profile data that are not only multidimensional but also exhibit extensive variability. This stands in stark contrast to Timer fluorescence data, which is inherently two-dimensional. This discrepancy poses significant analytical challenges in integrating and interpreting Tocky data.

We have identified key challenges that need to be addressed to solve the problem, not only concerning Timer data analysis but also extending to the broader scope of flow cytometric data analysis (Fig. 1D). First, data-oriented clustering needs to be improved in order to identify biologically meaningful cell clusters. Second, the dynamics of multidimensional marker profiles should be analysed in a way that is constrained by Timer fluorescence progression, so that temporal trajectories can be uncovered. Third, various cell clusters should be integrated in a meaningful manner to discover Timer-oriented developmental trajectories. Lastly, Timer data should be analysed quantitatively across identified developmental trajectories.

To address each of these challenges, we have developed several approaches, as shown schematically in Fig. 1E. The data preprocessing method involves normalising and applying trigonometric transformations to the Timer Blue and Red fluorescence data. This process is used to compute the ‘Timer Angle’, a measurement from the Blue axis within the Red-Blue plane that quantifies the elapsed time since TCR engagement in each cell (Bending et al., 2018a), and ‘Timer Intensity’, which correlates with signal strengths and accumulated transcriptional activities.

The workflow implemented in the new toolkit, ‘TockyDevelopment’, includes a novel clustering algorithm that integrates autofluorescence identification, PCA, and automatic clustering methods (‘PrecisionPCAClustering’; Fig. 1E). This enhancement significantly improves clustering accuracy by effectively handling autofluorescence noise, thereby facilitating data interpretation. Subsequently, a newly developed algorithm, ‘Tocky-Canonical Correspondence Analysis’ (Tocky-CCA), identifies interpretable dimensions of multidimensional marker expression data, constrained by Timer Angle and Intensity. As a variant of CCA fully adapted for flow cytometric analysis, Tocky-CCA enables the cross-analysis of three layers – marker profiles, single cells, and Timer progression (explanatory variables) – in a manner comparable to the CCA approach optimised for gene expression analysis (Ono et al., 2014, 2013). The Tocky-CCA solution will thus determine multidimensional relationships among developing cells based on Timer progression. To integrate cell clusters, a network approach, ‘Tocky-CCA Network’, is employed. This network, constructed using metrics determined in the Tocky-CCA solution space, facilitates visualisation to assist in the identification of key clusters. Furthermore, the implementation of a novel pathfinding algorithm, the ‘Dijkstra-Tocky Variant’, facilitates a data-driven identification of multiple developmental trajectories within developing T cells, highlighting distinct paths in cell differentiation and maturation. Lastly, a data categorisation method for Timer Angle, termed ‘Tocky Locus analysis’, is used to elucidate cell dynamics and marker expression dynamics, providing statistical outputs for trajectories and group comparisons.

### PrecisionPCAClustering enables efficient multidimensional clustering

Flow cytometry instrument settings are calibrated such that fluorescence-negative cells, defined as autofluorescence, span a wide range of numerical values, with true signals identified above a threshold value (Maecker and Trotter, 2006). This means that a substantial portion of expression data falls within the autofluorescence range and is biologically meaningless (Hulspas et al., 2009). Currently, it is not possible to automatically distinguish between autofluorescence and true signals (Verschoor et al., 2015), presenting a challenge in accurately identifying the temporal trajectory of developing cells. Therefore, developing an effective quantitative method for dissecting developmental trajectories using the Tocky system requires substantial improvements in the clustering method to reliably identify biologically meaningful clusters.

**Fig. 2.**
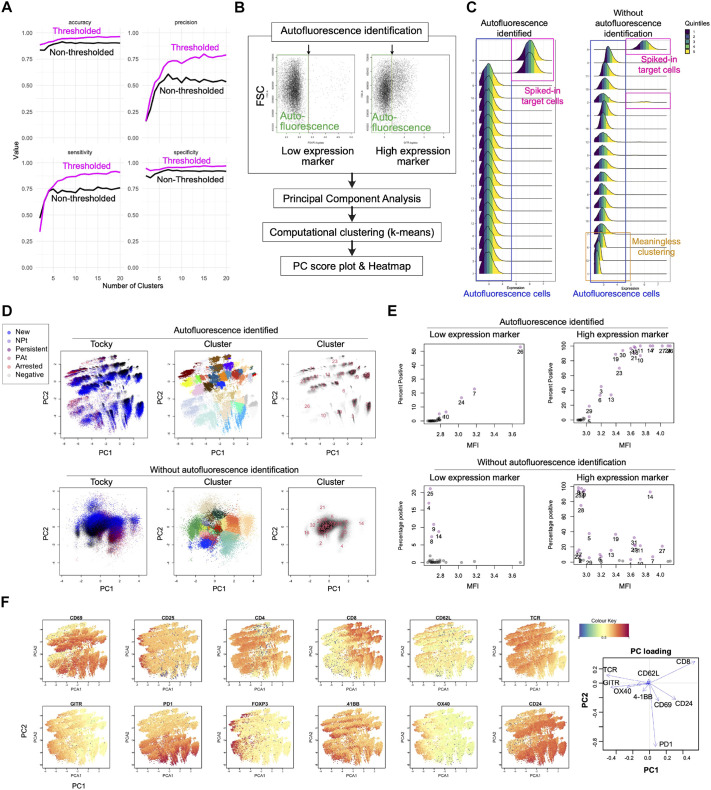
**Improved clustering method for flow cytometric data considering autofluorescence.** (A) Simulated data using Gaussian distributions with spiked-in target cells were analysed with the proposed clustering method using PCA and k-means. The effect of data thresholding of autofluorescence was analysed with respect to accuracy, precision, sensitivity, and specificity for identifying the target cells in enriched clusters, each of which had more than 20% target cells. (B) Flowchart of the new clustering method implemented in TockyDevelopment. Autofluorescence is determined by interactive windows following the standard practice of flow cytometric analysis for defining autofluorescence (negative cells). (C) The clustering of hybrid data with artificial spiked in cells with a bimodal distribution in a non-simulated, real flow cytometric dataset. High-dimensional flow cytometric data with and without autofluorescence identification were compared as histograms of the spiked-in data. Spiked-in target cells are shown by the magenta rectangles. Autofluorescence cells (marker-negative cells) are identified by blue rectangles. Meaningless clusters within the autofluorescence are highlighted by orange rectangle. (D,E) The clustering method was applied to the non-simulated dataset of developing thymocytes, identifying 32 clusters. Here, the high-dimensional flow cytometric data were analysed with and without autofluorescence identification, to test whether thresholding improved clustering efficiency. (D) PCA score plots are shown with or without heatmap of the Tocky locus (left), clusters shown by colour code (middle), and clusters shown by cluster identifier numbers (right). The cluster colour codes are not shown. (E) Mean Fluorescence Intensity (MFI) and the percentage of positive cells in each cluster for the indicated marker. Cluster identifiers are the same as in D. Clusters with more than 5% of target cells are highlighted in purple. (F) Left: Heatmap of the PCA plot of the data with autofluorescence identification in D, showing the expression level of the indicated marker or identified clusters. Right: PC loading of marker expression.

As we have previously reported, flow cytometry data can be effectively classified using k-means clustering combined with dimensional reduction through PCA (Fujii et al., 2016). Through simulations, we tested whether using a threshold to exclude autofluorescence enhances the enrichment of target cells within a limited number of clusters. Results show that the use of a threshold led to data being clustered more effectively, capturing spiked-in target cells across various cluster counts (Fig. 2A). This finding is important for the analysis of complex tissues with numerous distinct cell types, where a high number of clusters are necessary.

In our current computational package, we have implemented a hybrid approach that combines automatic clustering with manual definition of autofluorescence thresholds. This approach allows for sensitive detection of marker expression activation and downregulation. Initially, expression data are normalised based on a threshold that effectively distinguishes true signals from autofluorescence for each marker. PCA is then applied to these normalised data (Fig. 2B). To validate whether this new method prevents the clustering of cells within the autofluorescence range, we spiked artificial target cells into a flow cytometric dataset. The use of the threshold for autofluorescence successfully differentiated clusters solely composed of target cells from those composed of autofluorescent cells (Fig. 2C). Conversely, without exclusion of autofluorescence through identification and implementation of the threshold, cells within the autofluorescent range were incorrectly classified into several clusters based on sub-threshold expression levels.

We next used flow cytometric data from thymic T cells from Nr4a3-Tocky mice to analyse clusters obtained from a real dataset, to demonstrate that the new method produced visually distinct cell aggregates, each characterised by a unique Tocky locus (Fig. 2D). In the absence of autofluorescence identification and use of the threshold, cells failed to form distinct aggregates and instead clustered into a large, indistinct mass, indicating ineffective k-means classification. However, using PrecisionPCAClustering, this mass was subdivided into multiple clusters.

Inspection of these clusters further confirmed that autofluorescence identification and exclusion facilitated a more focused grouping of marker-positive cells, including those with both low and high expression levels. Notably, identification of autofluorescence revealed a clear correlation between the percentage of positive cells and their mean fluorescence intensity (MFI), a relationship that is absent without autofluorescence identification. This suggests that cells within the autofluorescence range can significantly distort classification efforts, impeding the accurate detection of genuinely positive cells. Moreover, identification of autofluorescence significantly improved the representation of cells expressing rare markers (Fig. 2E).

Finally, a heatmap was generated for each stained marker, showing that exclusion of autofluorescence facilitated the formation of distinct clusters with expression of distinct markers, while preserving gradual biological changes in marker expression. The heatmap in the PC score space corresponded directly to PC loadings (Fig. 2F). These findings collectively affirm that the new PCA-clustering algorithm with identification and exclusion of autofluorescence minimises the impact of autofluorescence, thereby distinguishing effectively between marker-positive and -negative cells, crucial for pinpointing distinct developmental stages in developing cells.

### Limitations in the existing method for analysing Tocky data

**Fig. 3.**
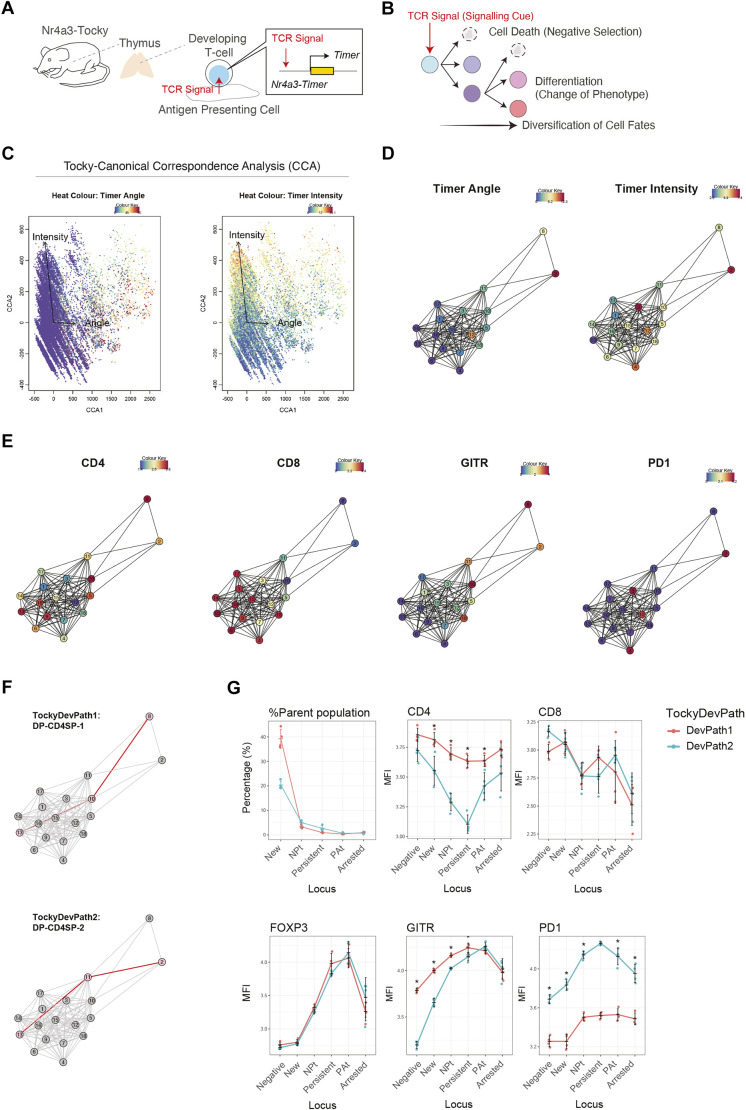
**Adaptation of CCA to high-dimensional flow cytometric Tocky data.** (A) The Nr4a3-Tocky system for the analysis of developing T-cells in the thymus. (B) The diversifying phenotypes and fates of developing T cells following T-cell receptor (TCR) signalling as a developmental cue. (C) Biplots of CCA with heatmap for Timer Angle (left) and Intensity (right). Each vector shows the correlated direction towards which cells increase either Angle or Intensity as indicated. (D,E) Tocky-CCA network analysis. Heat colours indicate Timer Angle and Intensity (D) and indicated marker expression (E). (F) Dijkstra-Tocky pathfinding, identifying two pathways for the two destinations, Clusters 8 and 2. (G) Tocky locus analysis for the developmental trajectories identified by the pathfinding. The percentage of cells in each Tocky locus among the parent population, after initiation of positive selection and CD69 upregulation in DP cells and the expression [mean fluorescence intensity (MFI)] of the markers indicated are shown in line charts for the two trajectories. The dataset included cells from four mice, and cells in the two trajectories were compared. *P<0.05, (Mann–Whitney test with *P*-value adjustment). Data are shown as mean±s.d.

The Tocky system facilitates the analysis of the temporal aspects of developing cells *in vivo*. In the thymus, developing T cells initiate their differentiation process in response to TCR signalling. Strong TCR signalling (or more precisely, cognate antigen signalling; Bending et al., 2018a) activates the Nr4a3-Timer transgene, subsequently inducing the expression of Timer protein (Fig. 3A). However, each developing thymocyte will receive different strengths and durations of TCR engagement at different instances in time, and so a major challenge arises from the variability in their individual characteristics, despite the overarching role of TCR signalling in guiding cellular development through major developmental stages. While TCR signalling drives T-cell maturation, individual T cells display unique, highly variable features, necessitating tailored analyses for different developmental trajectories within the thymic T-cell population (Fig. 3B).

To highlight the advantages of our novel toolkit, we first analysed a multidimensional flow cytometric dataset from Nr4a3-Tocky thymic T cells (Fig. S1). Traditionally, immunological studies employ a ‘manual gating approach’, which relies on multiple two- or one-dimensional plots to identify distinct cell populations using manually drawn polygons (gates) to ultimately isolate specific cell populations within a hierarchy of gates. As illustrated in Fig. S1, major thymic T-cell populations such as double-positive (DP; CD4 and CD8 positive) and CD4- and CD8-single-positive (SP) cells are first identified, and subsequently subdivided into subpopulations. The conventional categorisation of CD69 expression into high, intermediate, and low levels, based on its negative correlation with developmental progression in the thymus, produced some discernible Timer fluorescence patterns (Fig. S1A). However, the manual gating approach poses significant challenges for conducting comprehensive quantitative Tocky analysis. This method segments the data into discrete categories (populations), obscuring the continuous developmental pathways of T cells and hindering effective cross-analysis of multidimensional marker profiles with dynamic changes in Timer expression.

Dimensional reduction techniques such as UMAP cannot resolve this issue (Fig. S1B). Their inability to integrate multidimensional marker profiles with two-dimensional Timer fluorescence data without compromising the integrity of the temporal data captured by the Timer fluorescence results in a distortion or obscuring of crucial temporal information. This interference compromises the accuracy of tracing the temporal progression of individual thymocytes following initiation of TCR signal transduction, which is vital for understanding their maturation processes.

Furthermore, although the existing Tocky algorithm, which employs trigonometric transformation, provided insights into the gated populations and demonstrated a progressive increase in the mean values of Timer Angle (Fig. S1C), it falls short of fully capturing the dynamics of Timer Angle or addressing the limitations in cross-analysis between Timer data and multidimensional marker profiles.

### Identifying developmental trajectories in thymic T cells from Nr4a3-Tocky mice

Using the output from Tocky-CCA, we advanced our analysis to delineate the developmental trajectories and investigate the temporal dynamics of developing T cells. This analysis was conducted using the pipeline outlined in Fig. 1E, designed specifically to track and characterise the progression of T-cell maturation in Nr4a3-Tocky mice. As shown in Fig. 2D,E, T cells were successfully clustered by PrecisionPCAClustering.

#### Tocky-CCA and network analysis

To address the challenges of cross-analysing Timer progression with multidimensional marker profiles, we adapted CCA to high-dimensional flow cytometric data including Timer fluorescence (*Tocky-CCA*), as illustrated in Fig. 1E. Using Timer Angle and Intensity as explanatory variables, Tocky-CCA enables analysis of multidimensional marker profiles, guided by the dynamics of Timer progression. This approach allows for the integration of three layers of data – marker profiles, Timer progression, and individual cells – within a unified CCA framework. In this space, cells are positioned according to their similarities to one another, with their relationships further constrained by Timer progression.

Tocky-CCA was applied to the Nr4a3-Tocky thymus dataset, successfully aligning cells under the constraints of Timer Angle and Intensity (Fig. 3C). To further dissect the dynamics of T-cell development, we integrated cluster information from the Tocky-CCA results into a network model, referred to as the Tocky-Network. Specifically, the distances between clusters in the CCA space are reflected in the weights of the network edges, as detailed in the Materials and Methods section (Fig. 1E). This network construction highlighted Cluster 13 and adjacent clusters for their low values of Timer Angle and Timer Intensity, suggesting that these clusters consist of cells that have recently engaged in TCR signalling. These cells predominantly express both CD4 and CD8 markers, while exhibiting low levels of GITR (Tnfrsf18) and PD-1 (Pdcd1) expression, which are typical characteristics of DP cells. In contrast, Clusters 2 and 8 highly express CD4 and downregulate CD8, showing a high GITR expression, which is characteristic of CD4 single-positive (CD4-SP) cells.

#### Dijkstra-Tocky pathfinding and Tocky locus analysis

To identify developmental trajectories from DP cells to CD4-SP cells, the Dijkstra-Tocky method was employed (Fig. 1E, Fig. S2). This approach is a modified version of the traditional Dijkstra's pathfinding algorithm (Dijkstra, 1959), designed to search for the shortest path to the next unvisited node, with a unique constraint: transitions between pathway components are permissible only if the average Timer Angle of the destination cluster exceeds that of the originating cluster (Fig. 1E). This modification ensures that the algorithm captures cell developmental pathways with gradual changes in marker profiles, constrained by the increasing average Timer Angle (Fig. S2). Consequently, the Timer Angle serves as a critical metric in determining developmental trajectories through Timer progression.

Dijkstra-Tocky pathfinding identified a developmental path leading Cluster 13 cells to develop into Cluster 8 via Cluster 10 (designated as DP-CD4SP-1; Fig. 3F). Conversely, it revealed another path from Cluster 13 via Cluster 11 to Cluster 2 (designated as DP-CD4SP-2). Subsequently, Tocky locus analysis was employed to examine the dynamics of developing cells across these two trajectories (Fig. 3G). Intriguingly, DP-CD4SP-1 was characterised by sustained GITR expression across all Tocky loci, peaking at the Persistent locus. DP-CD4SP-2 featured consistently high PD-1 expression, with the highest levels observed at the Persistent locus. CD4 expression was transiently and moderately downregulated in DP-CD4SP-2 around the Persistent locus, while both trajectories showed a progressive decrease in CD8 expression. Similarly, Foxp3 expression was induced at the Persistent and Persistent-to-Arrested transitioning (PAt) loci, consistent with previous findings in CD4-SP cells (Bending et al., 2018a).

Collectively, this analysis pipeline revealed two distinct developmental trajectories for CD4-SP cells by the Nr4a3-Tocky system, elucidating previously unknown dynamics of key marker expression in a data-oriented manner.

### Elucidating Bcl2l11-dependent developmental trajectories in thymic T cells by deleting *Bcl2l11* in Nr4a3-Tocky

**Fig. 4.**
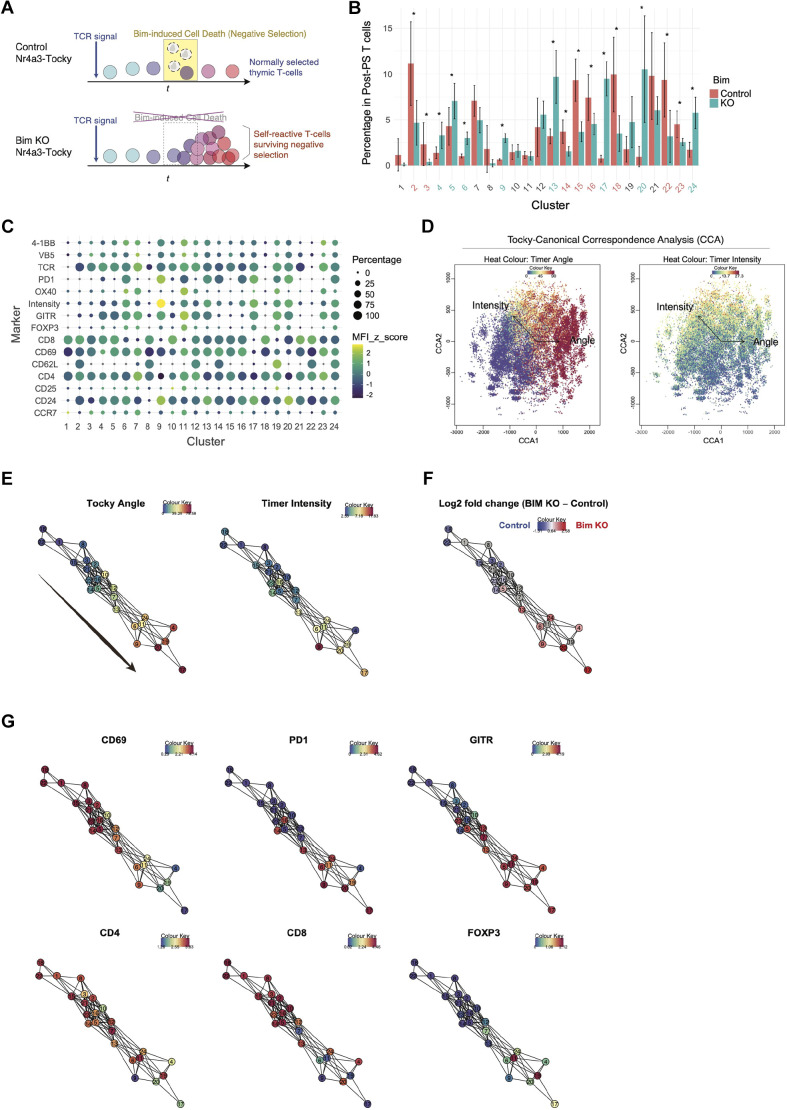
**Identifying developmental trajectories in thymic T cells from Nr4a3-Tocky mice.** (A) Schematic of the experimental setup to analyse the effects of *Bcl2l11* knockout on developing thymocytes. *Bcl2l11* deletion results in a lower rate of negative selection, which is expected to lead to accumulation of self-reactive T cells that are immediately deleted by negative selection in the presence of Bcl2l11. (B) Bar plot illustrating the percentage of cells post-positive selection (PS) within each cluster for two comparative groups. Numbers at the bottom of the bars are color-coded: green numbers signify clusters with a significant increase in the knockout (KO) group, whereas red numbers indicate clusters for which there is a significant increase in the control group. Data are shown as mean±s.d. The dataset includes control (*n*=6) and KO (*n*=8). *P<0.05 (Mann–Whitney test with *P*-value adjustment). (C) Dot plot displaying the percentage of cells that express the marker and the expression levels, represented by dot size and heat colour coding, respectively. (D) Biplot of Tocky-CCA output, showing developing T cells as dots and explanatory variables as arrows (Timer-Angle and -Intensity). Colours indicate the levels of Timer-Angle (left) or Timer-Intensity (right), as shown in the colour code. (E) The Tocky-CCA network is constructed using PCA clusters as nodes within the CCA space. Edges are determined by the distances between cluster centroids. Colours indicate the levels of Timer-Angle (left) or Timer-Intensity (right), as shown in the colour code. (F) Tocky network with heat colours for Log2 fold change of cell numbers in each cluster with Bim KO as the experimental group and Bim-sufficient as the control group. (G) Tocky network with heat colours for indicated marker expression.

Next, we applied the newly established clustering approach to the Nr4a3-Tocky Bim knockout (KO) dataset. This dataset was obtained by knocking-out *Bcl2l11* in the Nr4a3-Tocky background and analysing the thymi from both *Bcl2l11*^−/−^ Nr4a3-Tocky (Bim KO) and *Bcl2l11*^−/+^ Nr4a3-Tocky (Bim-sufficient, control) mice. *Bcl2l11*, a proapoptotic gene, plays a crucial role in thymic negative selection, which is predominantly driven by strong TCR signalling (Stritesky et al., 2013). Therefore, for simplicity, we use ‘Bim-sufficient’ as the control group to delineate Bcl2l11-dependent effects. We hypothesised that deletion of *Bcl2l11* in the Nr4a3-Tocky model would allow self-reactive T cells, which receive intense TCR signals, to survive longer than in controls, potentially altering their developmental dynamics (Fig. 4A).

PrecisionPCAClustering successfully identified eight clusters with higher percentages in the control and another eight clusters with higher percentages in the Bim KO (Fig. 4B). The dot plot, using percentages of clusters to determine the size of dots and MFI for heatmap colour, comprehensively visualises the marker profiles in the identified clusters (Fig. 4C).

To perform an integrative analysis of the clusters and analyse Bcl2l11-dependent effects on developing T cells, we proceeded with the Tocky analysis pipeline as above. The application of Tocky-CCA to the Bim KO Nr4a3-Tocky dataset successfully visualised the progression of Timer Angle and Intensity alongside multidimensional marker profiles at the single-cell level (Fig. 4D). The constructed Tocky-Network highlighted a progressive increase in Timer Angle from cluster 18 to cluster 17, with Timer Intensity mostly correlating with the progression of Timer Angle, as indicated by the directional arrow in Fig. 4E.

Intriguingly, comparison of cluster percentages between the Bim KO and the control group revealed that the Bim KO group accumulated developing T cells as Timer progressed (Fig. 4F). This accumulation indicates that the Nr4a3-Tocky system effectively captured the cellular dynamics due to *Bcl2l11* deletion and the resulting inefficient negative selection in T cells receiving strong TCR signals.

The Tocky-Network analysis also facilitated the visualisation of dynamic changes in marker profiles. Origin clusters such as 18, 22, 1, 8, and 15 showed high expression of CD69, CD4, and CD8, identifying them as DP cells undergoing repertoire selection. Clusters more mature in Timer expression exhibited reduced CD4 and/or CD8 expression and increased GITR expression (Fig. 4G). Thus, we identified three destination clusters based on the Tocky Network analysis shown in Fig. 4G: Cluster 19 (CD4-SP), Cluster 4 (CD8-SP), and Cluster 17 (DP-dull).

**Fig. 5.**
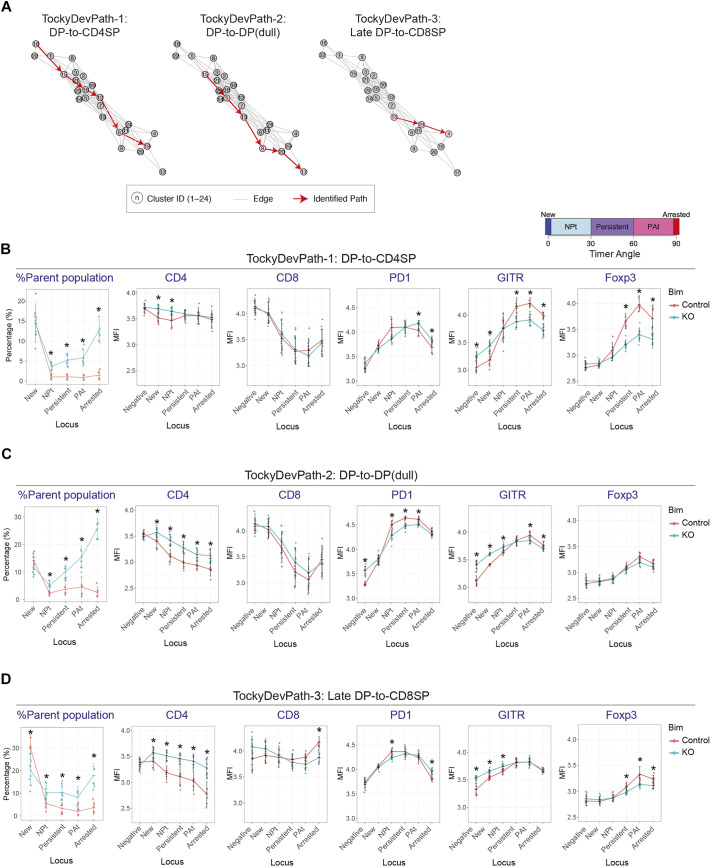
**Elucidation of Bim-dependent developmental trajectories in developing T cells by deleting *Bcl2l11* in Nr4a3-Tocky thymus.** (A) Dijktra-Tocky pathfinding was applied to the Nr4a3-Tocky Bim dataset, and identified three T-cell developmental trajectories within the CCA network, highlighted by bold pink edges and nodes: TockyDevPath-1, -2, and -3. (B-D) Line graphs showing the percentage of cells in each Tocky locus and their marker expression along defined Timer trajectories. The dataset included control (*n*=6) and KO (*n*=8). Data are shown as mean±s.d. *P<0.05 (Mann–Whitney test with *P*-value adjustment).

Using these clusters as destination, the Dijkstra-Tocky pathfinding was applied to the Tocky Network, identifying three distinct paths, ‘TockyDevPaths’ 1-3 (Fig. 5A). Given that the paths between clusters 18 and 15, and between clusters 15 and 13, are shared between TockyDevPaths 1 and 2, and TockyDevPaths 2 and 3, respectively, the overlapped paths have been omitted in TockyDevPaths 2 and 3. Consequently, clusters 15 and 13 are designated as the source clusters for TockyDevPaths 2 and 3, respectively. Subsequently, we conducted a Tocky locus analysis on the cells within these paths to better understand the time-dependent dynamics across the developmental trajectories (Fig. 5B-D).

TockyDevPath1 delineates the developmental trajectory from DP to CD4-SP (Fig. 5B). Within this pathway, the majority of control group cells were located in the New locus, whereas KO group cells progressively accumulated towards the Arrested locus, suggesting a Bcl2l11-dependent negative selection process. Notably, at the end of the Tocky locus, control cells significantly upregulated GITR and Foxp3, indicative of differentiation into Foxp3-expressing regulatory T cells, which are known to escape negative selection (Lee et al., 2012). In contrast, KO cells failed to upregulate GITR and Foxp3, instead showing elevated PD-1 expression towards the PAt and Arrested loci.

TockyDevPath2 illustrates the developmental trajectory from DP to DP-dull, marked by the downregulation of both CD4 and CD8 expression (Fig. 5C). Along this path, the Bim KO group exhibit a marked and progressive accumulation from the NPt to the Arrested loci, contrasting with control cells that are predominantly found in the New locus. This distribution suggests that the trajectory involves cells undergoing negative selection. Control cells display a progressive decrease in CD4 and CD8 expression, while markedly upregulating PD-1. Intriguingly, the surviving KO cells, which have endured severe selection pressures, do not downregulate CD4 as significantly as control cells and fail to increase PD-1 expression. Conversely, GITR expression is abnormally elevated in the KO group but does not show a peak at the PAt locus.

TockyDevPath3 delineates the developmental trajectory from late-stage DP to CD8-SP, characterised by downregulated CD4 expression with sustained CD8 expression (Fig. 5D). In this pathway, the KO group exhibits moderate accumulation from the NPt to the Arrested loci. Throughout this trajectory, differentiating T cells moderately upregulate PD-1 and GITR. Intriguingly, the KO group shows less PD-1 upregulation compared to the control group, whereas GITR expression is higher. Meanwhile, control cells are predominantly found in the New locus, with Foxp3 expression moderately induced in a minority of cells, a pattern not observed in the KO group.

Collectively, the novel pathfinding method applied to the Tocky-Network, which was constructed from the Tocky-CCA analysis of the Nr4a3-Tocky Bim KO dataset, successfully dissected the complex mix of developing thymic T cells. This approach identified distinct developmental trajectories that are controlled by Bcl2l11-mediated negative selection.

## DISCUSSION

We have developed TockyDevelopment, a novel software package designed for the multidimensional analysis of high-dimensional flow cytometric data from developing cells using the Tocky system. It elucidates developmental trajectories by integratively analysing multidimensional marker profiles and Timer progression using the Tocky-CCA algorithm and subsequent network analysis methods. This significant reduction in dimensionality is achieved through Tocky-CCA, which effectively identifies a constrained, reduced-dimensional space oriented by the progression of Timer. Subsequent refinement is performed by Dijkstra-Tocky pathfinding, which dissects this reduced dimensionality into distinct temporal trajectories guided by Timer progression. The analysis of the Nr4a3-Tocky Bim KO dataset underscores the biological significance of TockyDevelopment, and its ability to identify temporal trajectories. The potential for further development of this method includes identifying ‘trajectories’ as low-dimensional manifolds (i.e. partial spaces within a multidimensional space), thereby enhancing its utility in analysing complex data (Xu et al., 2023). Additionally, Tocky locus analysis, a feature of this package, further refines the temporal data according to Timer Angle progression, providing robust quantitative and statistical analysis. Collectively, the integrated techniques within TockyDevelopment provide a powerful tool for deciphering the temporally dynamic processes underlying cellular development, processes that are challenging to analyse with existing methods.

Tocky-CCA successfully elucidates marker profile dynamics across Timer progression with its capacity to perform a focused analysis on the multidimensional marker data that can be explained by the Timer fluorescence data. This is mathematically implemented as a regression analysis. Note that, in ter Braak's approach, CCA is adjusted for the row sums of the main input data and uses a χ^2^ matrix to balance the influence of different samples based on their total marker abundance (ter Braak, 1986). This adjustment assists the analysis of ecological data in which the total abundance can vary significantly between samples (van Dam et al., 2021). In addition, the χ^2^ metric helps in cases in which the absolute values are essential for understanding the data, such as in community composition studies for which the total count affects the interpretation (Muylaert et al., 2002). However, unlike the original ter Braak's CCA, Tocky-CCA does not use the sum of rows or the χ^2^ metric. This is because the absolute abundance or density of markers per cell does not carry biologically meaningful information in flow cytometric data. Thus, Tocky-CCA has been developed as a projection technique, aiming to analyse the relative expression levels of markers rather than their absolute quantities, and thus captures the dynamic changes of marker expression through Timer progression. Future studies could explore other multidimensional approaches to have a balanced analysis of multidimensional marker profiles and Timer fluorescence, such as multiple factor analysis (Kostov et al., 2013).

Importantly, the performance of the Dijkstra-Tocky pathfinder is influenced by the structure of the Tocky Network, and the selection of markers critically affects the output of Tocky-CCA and its network. This is because the outputs of multidimensional analysis are inherently dependent on the chosen set of variables (Wagner et al., 2016). Due to the limitations of flow cytometry, which restricts the number of surface proteins that can be analysed, integrating flow cytometric analysis of Tocky with other experimental techniques could be a promising solution. Previous studies investigating the dynamics of developing thymocytes have utilised various culture systems, including DP cell culture *in vitro* (Au-Yeung et al., 2014), fetal thymic organ culture (Solanki et al., 2018), and pseudotime analysis of single-cell RNA-seq data (Steier et al., 2023; Park et al., 2020). It is anticipated that *in vitro* culture could enhance the temporal resolution of Tocky analysis, and that a full integration of Tocky with single-cell RNA-seq could offer a powerful approach for analysing the temporal dynamics of developing cells.

Although further experimental investigations are necessary, the current study has unveiled previously unappreciated dynamics of developmental trajectories using the Nr4a3-Tocky system. Our analysis identified that wild-type Nr4a3-Tocky CD4-SP cells show GITR-prone and PD-1-biased development. While Wyss et al. identified a contrast between GITR^low^ PD-1^low^ and GITR^high^ PD-1^high^ CD4 SP cells (Wyss et al., 2016), the differential developmental pathways have not been fully described to our knowledge. Notably, GITR is induced in CD4-SP cells before they express Foxp3 (Ono et al., 2006). Given the observed reduction in Foxp3 and GITR expression in Bim KO mice (c.f. Fig. 5), it is evident that the Bcl2l11-dependent CD4 SP developmental trajectory is finely controlled by factors including GITR, PD-1, and Foxp3. Future investigations employing Foxp3-Tocky (Bending et al., 2018a,b) should further elucidate these intricate developmental mechanisms.

Furthermore, the TockyDevPath-2, as illustrated in Fig. 5, captures the development of DP cells into PD-1^high^ DP cells, which McDonald et al. (2015) identified as cross-reactive T cells with Class-I and Class-II MHCs primarily deleted through negative selection. The marked accumulation of these cells in Bim KO across the TockyDevPath-2 Timer Angle (Fig. 5E) supports that this trajectory is strictly controlled by Bcl2l11. Conversely, TockyDevPath-3 illustrates the development into CD8-SP cells, albeit less controlled by Bcl2l11. Notably, PD-1 and GITR are dynamically regulated across all three Bcl2l11-dependent developmental trajectories, underscoring their dynamic roles during negative selection.

While the current study effectively addresses cell death controlled by Bcl2l11, it may account for cells that undergo apoptosis or necrosis during development independently of Bcl2l11-induced pathways. A significant challenge arises because dying cells are promptly removed from the system, and the use of dead cell stains in flow cytometry excludes transitional cells from analysis. To overcome these issues, future enhancements are planned to integrate our existing Tocky trajectory analysis with single-cell RNA-seq. This combination will allow for a detailed analysis of gene expression profiles alongside Tocky trajectory data, providing a more nuanced understanding of thymic cell fates. Such advancements are expected to enrich our comprehension of T-cell maturation by capturing a broader spectrum of cellular states and transitions, thereby addressing important gaps in the current methodology.

In conclusion, this study enhances the efficacy of the Tocky system through our novel toolkit, which is designed for analysing the temporal dynamics of developing cells via multidimensional flow cytometric data. With the expanding capabilities of the Tocky system (Reda et al., 2024 preprint), our novel toolkit not only enhances our understanding of complex cellular dynamics but also offers unique solutions to previously challenging problems in developmental cell biology, molecular biology, immunology, and related research fields. This toolkit is supported by comprehensive documentation that facilitates its use among researchers with experience using R packages. In the future, we may simplify the toolkit's operation to extend its usability to a broader research audience and reduce reliance on computational expertise.

## MATERIALS AND METHODS

### Mice and flow cytometry

Nr4a3-Tocky was generated by the Ono lab and carried *Foxp3^IRES-EGFP^* (B6.Cg-*Foxp3^tm1.1Mal^*/J; The Jackson Laboratory, 018628) as previously described (Bending et al., 2018a). Briefly, using a knock-in knockout approach, the first coding exon of the *Nr4a3* gene was replaced with the Fluorescent Timer gene *Fast-FT*, an mCherry mutant (Subach et al., 2009). Nr4a3-Tocky Bim KO mice were obtained by crossbreeding Nr4a3-Tocky mice with Bim KO mice (B6.129S1-*Bcl2l11^tm1.1Ast/J^*; The Jackson Laboratory, 004525). All animal work was performed in line with UK Home Office Animal Scientific Procedures Act 1986 at Imperial College London.

The flow cytometric datasets were generated using thymi from 4-week-old mice, both males and females, from either the Nr4a3-Tocky colony or the Nr4a3-Bim KO colony. Briefly, single-cell suspensions were first stained with a fluorescent viability dye (eFluor 780, 65-0865-14, Invitrogen) and Fc-block (14-0161-82, eBioscience). This was followed by staining for non-T-cell markers using the following biotinylated antibodies: CD11b (M1/70, 101211), CD11c (N418, 117310), TCR-γδ (eBioGL3, 13-5711-82), Ly6G/C (R86-8C5, 13-5931-82), TER-119 (TER-119, 13-5921-82), and CD19 (eBio1D3, 17-0193-82) (all from Thermo Fisher Scientific) and NK1.1 (PK136, BioLegend, 13-5941-82). Subsequently, the cells were stained with the following fluorochrome-conjugated antibodies: CD4 (GK1.5, BUV395, 565974), CD8α (53-6.7, BUV737, 612759), GITR (DTA-1, BV650, 740618), and CD62L (MEL-14, BUV805, 741924) from BD; TCRβ (H57-597, BV605, 109241), PD-1 (29F.1A12, BV711, 135231), CCR7 (4B12, BV785, 120127), OX40 (OX-86, PE/Cy7, 119415), CD25 (PC61, Alexa Fluor 700, 102024), and Streptavidin APC-Cy7 (405208), 4-1BB (17B5, PE, 106105), CD24 (M1/69, PerCP-Cy5.5, 101823) from BioLegend; Vβ5.1/5.2 (MR9-4, PerCP-eF710, #46-5796-82) and CD69 (H1.2F3, PE and APC, #17-0691-82) from Thermo Fisher Scientific.

### Data cleaning and gating

FlowJo was used for cleaning data by gating live lymphocytes, excluding the lineage marker-positive cells and dead cells, and for the demonstration of the common and traditional gating approach. In addition, t-distributed stochastic neighbour embedding plots were generated by a FlowJo plugin. Live thymocytes expressing either CD69^+^ or TCRβ^+^ lymphocytes were identified using FlowJo and data were exported as csv files for downstream analysis, which was all performed using R (4.3.1).

### Tocky Angle and Intensity

The algorithm for calculating Tocky Angle and Intensity was previously reported (Bending et al., 2018a). Wild-type cells were used to determine the thresholds for Timer fluorescence. Time Angle and Timer Intensity were computed by trigonometric data transformation of blue and red fluorescence, as previously described (Bending et al., 2018a). Briefly, after normalisation of Timer Blue and Red fluorescence, Timer Angle was defined by the angle from the Blue axis in the Blue-Red plane. Timer Intensity was defined as the distance (norm) of each cell from the origin.

### Dimensional reduction and clustering

A threshold for autofluorescence was established for each marker expression by either a histogram or a two-dimensional plot, which combines marker expression with forward scatter. This process is implemented in the TockyDevelopment package through interactive sessions. PCA was applied to the thresholded data and PC scores were used for k-means clustering as previously reported (Fujii et al., 2016). UMAP used the R package ‘umap’ (R package version 0.2.10.0. https://CRAN.R-project.org/package=umap).

### Tocky-CCA

Tocky-CCA is a variant of CCA, which was originally developed by ter Braak and standardises main matrix using a χ^2^ metric (ter Braak, 1986). Adapting the algorithm to flow cytometric data, Tocky-CCA focuses on the direct relationship between the standardised explanatory variables Timer Angle and Intensity **Z** (n×2), which is normalised column-wise by dividing each column with its standard deviation, and multidimensional marker profile data **S** (n×p), which is scaled and centred column-wise such that each marker (column in **S**) has a mean of 0 and is standardised to have a standard deviation of 1. Note that n is the number of cells and p is the number of markers. To perform Tocky-CCA, **S** is projected onto the **Z** using the projection matrix **P**=**Z**[t(**Z**)**Z**]^−1^. As [t(**Z**)**Z**]^−1^ calculates the cross-correlations within these Timer Angle and Intensity measurements for weighing the contributions of each of the time-related variable in subsequent projections, **P** is the coefficient used to project the main multidimensional marker profile data onto the column space of **Z**, and these coefficients will adjust how the multidimensional marker profiles in **S** are influenced by the temporal data in **Z***.*

The Timer-constrained marker profile data uses the projection coefficient **P** to weigh the cross-product t(**Z**) **S**, i.e. S*=P t(**Z**)**S**. As t(**Z**)**S** calculates how each of Timer Angle and Intensity in **Z** correlates with each marker in **S**, it quantifies the associations between the temporal aspects of cell development and the expression levels of various markers. The result, S*, is the regression coefficient that best describes the dependencies of the marker profile **S** on the timer fluorescence **Z**. Thus, S* effectively models how changes in Timer Angle and Intensity predict changes in cellular markers. Then, CCA weighted average (wa) scores for single cells are calculated using *X_ij_* from the explanatory variable Timer Angle and Intensity and the marker variables **Y** (p×2)=**SV**:

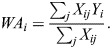
Thus, wa scores will provide a summarised measure of overall expression of all marker expression data in each single cell, taking into account the influence of the progression of Timer Angle and Intensity.

### Tocky network analysis

The wa score of CCA was used for temporal network analysis. Adjacent matrix was produced using CCA wa score for clusters. The lowest percentile that allows connection of all clusters by at least an edge was chosen for each network. The visualisation of the network was assisted by the CRAN package *igraph* (Csardi and Nepusz, 2006).

### The Dijkstra-Tocky variant for pathfinding in the Tocky network

To analyse Tocky Network data from developing thymocytes, a modified Dijkstra's algorithm has been established. The novel Dijkstra-Tocky algorithm modifies the conventional Dijkstra's algorithm (Dijkstra, 1959) to include a constraint that only allows progression from one pathway component to another if the Timer Angle of the destination component is greater than that of the originating component. The Dijkstra-Tocky algorithm adapts the classic Dijkstra's algorithm, incorporating the Tocky approaches to capture the temporal progression of cell development using the Tocky approach. Here, Timer Angle serves as a quantifiable measure of the temporal sequence, ensuring that the pathfinding algorithm respects the necessary temporal order of events. This adaptation effectively transforms the analysis into a semi-directed graph problem, where the directionality is dictated by the Timer Angle values, thus introducing a ‘time-respecting’ pathfinding mechanism.

The outline of the Dijkstra-Tocky algorithm is as follows. (1) Initialisation: The variant initialises distances from the source node (representing the initiation of the immune response) with the source set to zero and all others to infinity, akin to traditional Dijkstra's setup. (2) Pathfinding under constraints: Unlike traditional Dijkstra's algorithm, the Dijkstra-Tocky variant iterates over the graph's vertices, updating distances only if the Timer Angle constraint is satisfied, thereby ensuring the algorithm adheres to the developmental sequence of events.

### Statistics and visualisation

Base functions in R were used to perform non-parametric Mann–Whitney *U*-tests with *P*-value adjustment using the Benjamini–Hochberg method (Benjamini and Hochberg, 1995). Visualisation was assisted by the CRAN packages ‘ggplot2’ (Wickham, 2016) and ‘ggridges’ (R package version 0.5.6. https://CRAN.R-project.org/package=ggridges). A heatmap was produced for the expression of markers in each node using the CRAN package ‘RColorBrewer’ (R package version 1.1-3. https://CRAN.R-project.org/package=RColorBrewer).

## Supplementary Material



10.1242/develop.204255_sup1 Supplementary information
