# Supplementary material for: A multidimensional toolkit for elucidating temporal trajectories in cell development in vivo
Source: Development. 2024 Dec 18;151(24):dev204255. doi: 10.1242/dev.204255 (PMC11701510; doi:10.1242/dev.204255)
Supplement: Supplementary information [file develop-151-204255-s1.pdf]

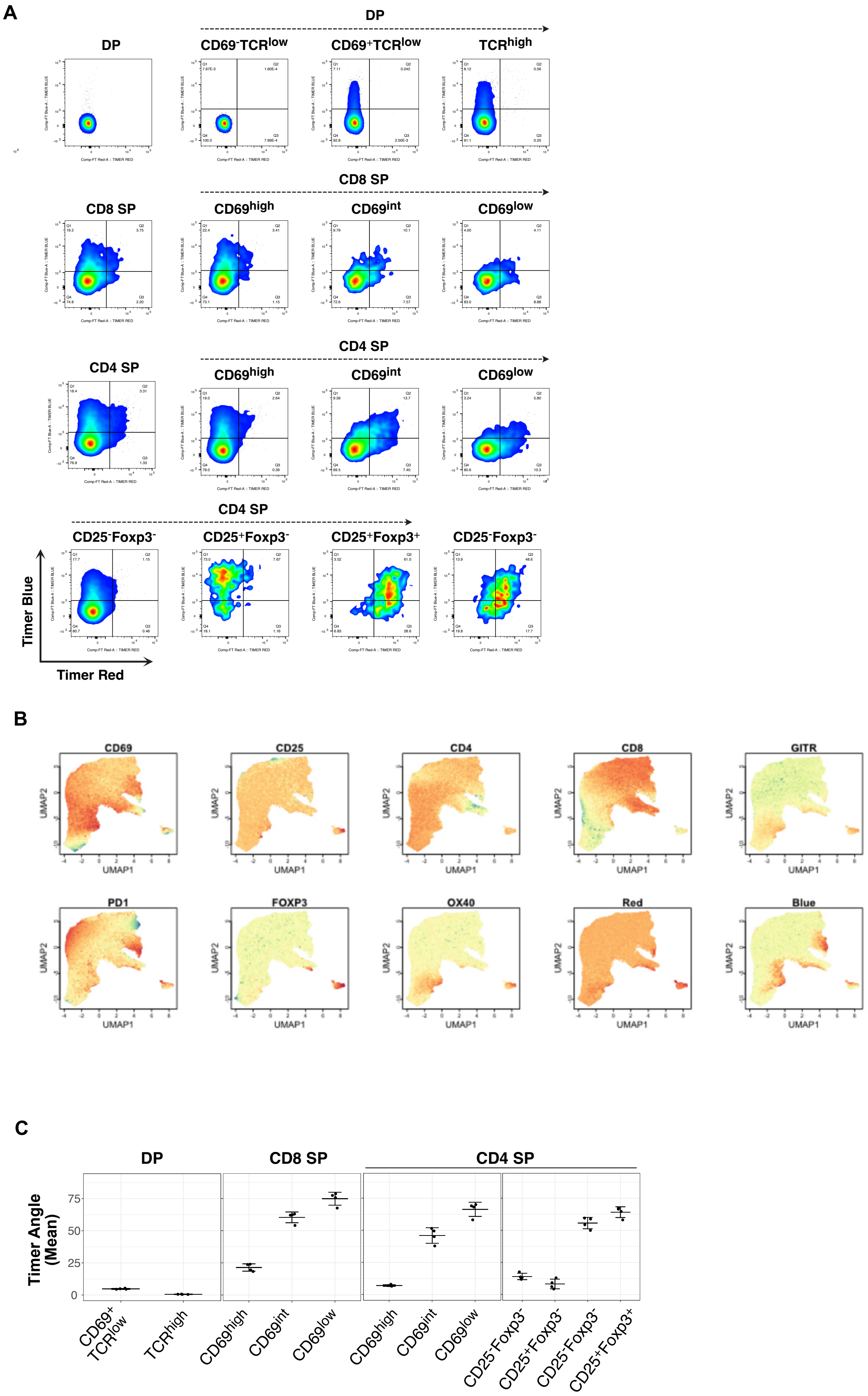

Development • Supplementary information

Fig. S1. Limitations of the existing Tocky methods for analysis of developing thymocytes

(A) Schematic figure showing the current standard approach for flow cytometric analysis and the existing Tocky analysis methods (Tocky 1.0). The analysis can trifurcate into three branches of analysis, including (1) successive manual gating for population identification, (2) Tocky trigonometric transformation, and (3) multidimensional analysis (e.g. UMAP, t-SNE).

(B) Representative flow cytometric data using the conventional approach to analyse data from Nr4a3-Tocky thymocytes. Double Positive (DP) and Single Positive (SP) populations and their subpopulations were identified using the indicated marker profiles

(C) An existing Tocky analysis method showing the mean Timer Angle of each sample is plotted after trigonometric transformation of Timer fluorescence data. The dataset included cells from four Nr4a3-Tocky mice.

Development • Supplementary information

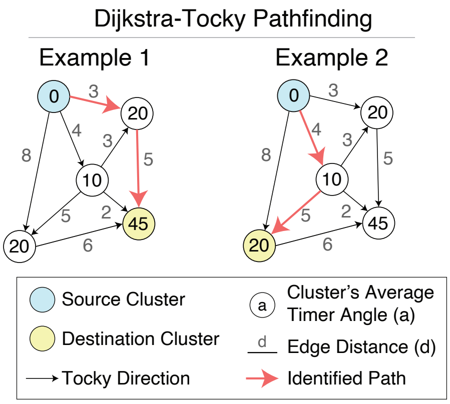

Fig. S2. Dijkstra-Tocky Pathfinding Algorithm

Simple examples are provided to illustrate how the Dijkstra-Tocky Pathfinding Algorithm identifies paths, demonstrating the specific conditions imposed by the algorithm.

Development • Supplementary information
