# Supplementary material for: A multidimensional toolkit for elucidating temporal trajectories in cell development in vivo
Source: Development. 2024 Dec 18;151(24):dev204255. doi: 10.1242/dev.204255 (PMC11701510; doi:10.1242/dev.204255)
Supplement: Supplementary file 1 [file develop-151-204255_review_history.pdf]

A multidimensional toolkit for elucidating temporal trajectories in cell development *in vivo*

Masahiro Ono and Tessa Crompton
DOI: 10.1242/dev.204255

Editor: Hanna Mikkola

Review timeline

Original submission:	16 July 2024
Editorial decision:	1 November 2024
First revision received:	8 November 2024
Accepted:	17 November 2024

Original submission

First decision letter

MS ID#: DEVELOP/2024/204255

MS TITLE: A Multidimensional Toolkit for Elucidating Temporal Trajectories in Cell Development In Vivo

AUTHORS: Masahiro Ono; Tessa Crompton

I have now received the referees reports on the above manuscript, and have reached a decision. The referees' comments are appended below, or you can access them online: please go to.

The overall evaluation is positive and we would like to publish a revised manuscript in Development, provided that the referees' comments can be satisfactorily addressed. Please attend to all of the reviewers' comments in your revised manuscript and detail them in your point-by-point response. If you do not agree with any of their criticisms or suggestions explain clearly why this is so. If it would be helpful, you are welcome to contact us to discuss your revision in greater detail.

Reviewer 1

Advance summary and potential significance to field

Understanding the factors that regulate the differentiation and development of T cells is one of the most fascinating area of immunological research because of its complexity and its relevance to pathophysiology. These investigations have always been very challenging because of the inherent plasticity of phenotype of T cells and the facts that both development and differentation are very dynamic processes that might occur at different times in different cells. The TockyDevelopment toolkit presented in this paper is specifically designed to address the challenge of analyzing asynchronous cell populations. Indeed, this innovative experimental system, takes this into account by assessing the following:

Timer Fluorescence System: A fluorescent timer protein changes color over time from blue to red, allowing each cell to carry its own "timestamp" of when it received a signaling cue (in this case, TCR signaling).
Timer Angle and Intensity: The trigonometric transformation of the Timer Blue and Red fluorescence data are used to calculate Timer Angle and Timer Intensity for each cell. Timer Angle

© 2024. Published by The Company of Biologists under the terms of the Creative Commons Attribution License (https://creativecommons.org/licenses/by/4.0/).

serves as a measure of time since signal activation, while Timer Intensity correlates with signal strength.
These two parameters allow for analysis of cells at different stages of development within the same sample and inherently account for the asynchronous nature of cell development in vivo.
The proposed Tocky-CCA adapted algorithm is next used for the analysis of how marker expression changes over time across the asynchronous population while the Tocky Locus Analysis allows the classification of cells into different "loci" based on their Timer Angle (New, NPt, Persistent, PAt, Arrested).
By integrating all these data , the TockyDevelopment toolkit can effectively provide insights into T cell developmental trajectories and dynamics that would be challenging to obtain with other methods.

Comments for the author

I think this is a very powerful and interesting experimental model that can be used for a wide range of applications and study. I do not have any question in terms of experimental data and overall idea behind this study. My only request is for some further clarifications in the text:

1- The authors state that the source code for the TockyDevelopment package will be freely available on GitHub upon publication, fulfilling the requirement of making the resource available with minimal restrictions. However, the system is based on the use of several sophisticated algorithms, which may require significant computational resources and expertise to implement and interpret. Are there any plans to develop a user-friendly interfaces and optimizing computational efficiency could make the toolkit more accessible to a broader range of researchers? If so, could the authors provide in the text some details about this?

2- I am not sure if this is correct but the current analysis may not fully account for cells that die during the developmental process, potentially biasing the observed trajectories. If this is correct, is there any way to Incorporate methods to detect and account for cell death that could provide a more complete picture of developmental dynamics?

First revision

Author response to reviewers' comments

Point-by-point Response

1- The authors state that the source code for the TockyDevelopment package will be freely available on GitHub upon publication, fulfilling the requirement of making the resource available with minimal restrictions. However, the system is based on the use of several sophisticated algorithms, which may require significant computational resources and expertise to implement and interpret. Are there any plans to develop a user-friendly interfaces and optimizing computational efficiency could make the toolkit more accessible to a broader range of researchers? If so, could the authors provide in the text some details about this?

Response: We appreciate your suggestion concerning the development of user-friendly interfaces to enhance the accessibility of the TockyDevelopment toolkit. Our package will be provided with comprehensive documentation designed to support users who possess some experience in using R packages. This will facilitate the implementation and interpretation of our sophisticated algorithms. In the future we may simplify the toolkit's usability, to make it accessible to a broader audience and reduce the need for extensive computational expertise. These points have been added to our revised manuscript on page 18.

2- I am not sure if this is correct but the current analysis may not fully account for cells that die during the developmental process, potentially biasing the observed trajectories. If this is correct, is there any way to Incorporate methods to detect and account for cell death that could provide a more complete picture of developmental dynamics?

© 2024. Published by The Company of Biologists under the terms of the Creative Commons Attribution License (https://creativecommons.org/licenses/by/4.0/).

Response: Regarding your concern about the exclusion of cells that die during the developmental process, we recognise that this represents a potential limitation in our current analysis framework. Even using the Bim KO approach, some cells may die through a Bim-independent mechanism, and it is experimentally challenging to analyse dead cells directly using our current methods. However, we are exploring future studies that will integrate single-cell RNA sequencing (scRNA-seq) with the Tocky trajectory method in the current report. This integration is anticipated to more effectively address the dynamics of cells, including dying cells, and provide a more complete picture of developmental trajectories by capturing a wider range of cellular states and transitions. These points have been included in our revised manuscript on page 17.

Appendix: Summary of changes made to the manuscript to meet the criteria outlined in the journal's instructions and checklist

- Abstract: The abstract has been condensed to 179 words.
- Acknowledgements: This section has been updated to include all relevant information.
- Materials and Methods:
- Mice and Flow Cytometry (Page 19): Detailed genotype information and antibody details have been added to the text.
- Figure Legends (Pages 26, 27, and 28): The number of mice used in the experiments and the statistical methods employed have been included in relevant parts.
- Citations: Citations have been updated to include key references for the computational packages used.
- Figure 1: The logo of TockyDevelopment has been removed to avoid potential copyright issues.

<u>Second decision letter</u>

MS ID#: DEVELOP/2024/204255

MS TITLE: A Multidimensional Toolkit for Elucidating Temporal Trajectories in Cell Development In Vivo

AUTHORS: Masahiro Ono; Tessa Crompton
ARTICLE TYPE: Techniques and Resources Article

I am happy to tell you that your manuscript has been accepted for publication in Development, pending our standard publication integrity checks.

© 2024. Published by The Company of Biologists under the terms of the Creative Commons Attribution License (https://creativecommons.org/licenses/by/4.0/).
